# Serum triglycerides as a risk factor for cardiovascular diseases in type 2 diabetes mellitus: a systematic review and meta-analysis of prospective studies

**DOI:** 10.1186/s12933-019-0851-z

**Published:** 2019-04-15

**Authors:** Xiaofeng Ye, Wen Kong, Mohammad Ishraq Zafar, Lu-Lu Chen

**Affiliations:** Department of Endocrinology, Union Hospital, Tongji Medical College, Huazhong University of Science and Technology, Wuhan, 430022 China

**Keywords:** Triglycerides, Type 2 diabetes mellitus, Cardiovascular diseases, Meta-analysis

## Abstract

**Objective:**

The importance of triglycerides (TG) level as a risk factor for cardiovascular diseases (CVD) has been extensively investigated in the general population; however, their relationship in patients with type 2 diabetes mellitus (T2DM) is uncertain. We aimed to assess the association of TG with CVD in T2DM individuals.

**Research design and methods:**

We searched bibliographic databases for studies published until June 2018, reporting on the relationship between TG and CVD in T2DM people. A random-effects model with inverse variance weighting was used to compute pooled estimates of the most fully adjusted risk ratios (RR) and corresponding 95% confidence intervals (CI) according to TG categories, unit TG, and logarithm (log) of TG for CVD.

**Results:**

A total of 31 studies were included, involving 132,044 T2DM patients with 10,733 incident cardiovascular events. The pooled RR (95% CI) of CVD for an increase in baseline TG, log TG by 1-mmol/l and categorized in the highest vs. the lowest TG in T2DM were 1.06 (1.02, 1.09), 1.30 (1.18, 1.42) and 1.30 (1.16, 1.46), corresponding to a CVD risk increase of 6%, 30% and 30%, respectively. The pooled RR (95% CI) of CVD for per 1-mmol/L TG increment in eight studies and TG categories in three studies were 1.03 (0.98, 1.08) and 1.39 (0.92, 2.1) in T2DM patients adjusted for other lipids parameter, respectively.

**Conclusions:**

In T2DM patients, an elevated triglyceride level cannot serve as an independent marker for an increased risk of cardiovascular events, but still, the higher serum TG levels tend to be associated with increased risks of CVD.

**Electronic supplementary material:**

The online version of this article (10.1186/s12933-019-0851-z) contains supplementary material, which is available to authorized users.

## Introduction

Cardiovascular diseases (CVD) are becoming increasingly frequent and associated with a high incidence of disability, and death. CVD are the first cause of mortality in the world, representing 31.5% of all global deaths. In 2015, 17.7 million people died by CVD according to the World Health Organization (WHO) [1]. Prevention of CVD through the control of risk factors is a priority in most developed countries [2].

The prevalence of T2DM is rising at an alarming rate globally. T2DM as a common and severe condition has posed a significant burden on patients, their families, and health care systems. T2DM is a significant risk factor for CVD, such as coronary heart disease (CHD), stroke, and peripheral arterial disease. Patients with T2DM have a 2–4 times higher risk of CVD incidence than those without diabetes [3]. CVD is the leading cause of death among individuals with T2DM, accounting for approximately 70% of death [4, 5].

It is well documented that elevated blood pressure, hyperglycemia and low-density lipoprotein cholesterol (LDL-C) abnormalities are vital contributors to the risk of CVD in individuals with diabetes [6–8]. Several meta-analyses and reviews have highlighted the relationship between TG level, and risk of CVD among the general population, an increase in the TG concentration is exposed to the higher risk of CVD events [9–11]. However, the association of elevated TG level and the risk of CVD in type 2 diabetic population is still not conclusive. Some studies found that there is no association between TG and the risk of CVD among T2DM [12, 13], whereas some studies found the higher rate of CVD incidence in diabetic individuals [14, 15]. In randomized trials, patients used medications that reduce triglyceride levels also had different results in CVD risk. Clinical trials of agents that lower TG, specifically fenofibrate and niacin, have failed to demonstrate a reduction in CVD outcomes when administered in addition to appropriate medical therapy [16, 17]. Recent studies of n − 3 fatty acid products have not shown a benefit in patients receiving statin therapy [18–20]. However, the Reduction of Cardiovascular Events with Icosapent Ethyl-Intervention Trial (REDUCE-IT) study indicated that the risk of major ischemic events, including cardiovascular death, was significantly lower with icosapent ethyl compared to placebo in patients with elevated triglyceride levels [21].

All these studies have yielded conflicting results. To our knowledge, there has been no systematic analysis of these evidence to date. Thus, we performed a systematic review and meta-analysis on the available studies to evaluate the relationship between circulating TG and CVD in patients with T2DM.

## Methods

### Data sources and searches

The current systematic review and meta-analysis was conducted following the guidelines of Meta-Analysis of Observational Studies in Epidemiology (MOOSE) [22]. Two authors (XFY & WK) performed the systematic searches on bibliographic databases (PubMed, EMBASE, Cochrane Library, clinicaltrails.gov, Web of Science, China National Knowledge Infrastructure (CNKI), and Wanfang Databases) for articles published till June 2018 without any language restrictions. The keywords applied to retrieve potential articles for inclusion were: ‘triglycerides’, ‘TG’, ‘lipids’, ‘cardiovascular events’, ‘cardiovascular outcomes’, ‘vascular disease’, ‘coronary heart disease’, ‘stroke’, ‘cerebrovascular disease’, ‘type 2 diabetes’, ‘non-insulin-dependent diabetes mellitus’, ‘T2DM’ in combination of medical subject heading (Mesh) and text. Reference lists of the retrieved articles were also scanned manually for identifying additional relevant studies.

### Study selection

The prospective studies were included if they reported the hazard ratio (HR), or equivalent, and its 95% CI, on the relationship between the TG and CVD in T2DM. Where multiple publications reported associations from the same cohort, we included the study with the longest follow-up period or one with the largest sample size. We excluded the studies that used surgical intervention for the treatment of diabetes or studies those had a follow-up period of fewer than 12 months.

### Data extraction and quality assessment

Two authors (XFY and WK) independently performed the data abstraction of the following information from eligible articles: article title; author details; year of journal publication; study location; study design; study size; follow-up duration; age at baseline; sex distribution; duration of diabetes; endpoint definition and ascertainment; study outcomes; HR and 95% CI of TG for CVD.

The quality of included studies was assessed by two authors (XFY and WK) by New Castle–Ottawa Scale (NOS) [23]. Any disagreement related to data procurement or study quality evaluation was resolved either by mutual consensus or by referring to a third author (MIZ).

### Data synthesis and analysis

For each outcome, the association between TG level and CVD risk (HR or RR or OR) and it’s 95% CI was converted into RR. The reported RR was classified into three types, categorical TG, continuous TG or log TG. Meta-analysis of categorical risk estimates compared RR for the highest TG group vs. the lowest TG group [24]. Studies varied considerably in the thresholds used in categorization of TG. The continuous RR was reported as per unit or standard deviation change in TG. All continuous RR were rescaled to represent per 1 mmol/l increase, by applying the method discussed by Shi and Copas [25]. The log TG RR was also rescaled to per 1 mmol/l increase.

The DerSimonian and Laird random-effects model of inverse variance methods were applied to estimate the pooled risks and 95% CI [26]. Unless otherwise stated, we used the most fully adjusted RR from each study. Statistical heterogeneity was evaluated by the I^2^ statistic; the I^2^ values of 25% to < 50%, 50% to < 75%, and ≥ 75% represent as small, medium, and large degree of heterogeneity, respectively [27]. We drew the funnel plots to visualize the possible publication bias. Theoretically, the minimum number of studies required to draw plot is ten. The potential publication bias was quantified by using Begg’s and Egger’s test [28, 29], and a p-value of less than 0.05 was considered statistically significant.

We also performed the subgroup analyses to identify the possible sources of heterogeneity and determined their culpable effects in different subgroups. Pre-defined subgroup analyses were conducted by adjusting lipid levels, glycemic levels, blood pressure, baseline age (≥ 65 years old or not), gender (male, female or mixed population), duration of follow-up (≥ 5 years or not), geographic location (Europe/America or Asia–Pacific), free CVD history at baseline (Yes or No), baseline glycated hemoglobin (HbA1c) (≥ 7% or not), renal function (estimated glomerular filtration rate (eGFR) ≥ 60 min/ml/1.73 m^2^ or not), and categorical (categorical two or not). All analyses were performed using Stata version 14.0. A p-value < 0.05 was considered significant.

## Results

### Search results

The article selection process is depicted in Additional file 1: Figure S1. A total of 5282 articles were retrieved from the preliminary search. Screening of these articles (title/abstract) leads to the exclusion of 4981 articles, as they were duplicates, irrelevant to the study context and study types (meta-analysis or review articles), a total of 301 articles were subjected to further evaluation. On further screening, we found a total of 31 eligible studies including one article identified from manual search were included in the quantitative analysis.

### Study characteristics

The characteristics of the included studies are shown in Additional file 1: Table S1. Overall, most of the information was available for 132,044 T2DM patients with 10,733 incident cardiovascular events. The follow-up length varied between 1 and 13 years. The mean age was 59.8 years, and 56.2% of the total study population was male. Most of the study used fasting TG and only one study used postprandial TG. We included randomized control and cohort studies. Five studies were conducted in the United States, ten in Europe, two in Australia, two in New Zealand, eleven in Asia, and one in Brazil. The adjustment of factors included were age, sex, duration of diabetes, insulin use, hypertension, smoking, alcohol consumption, and plasma lipid levels, etc. Most of the studies achieved a moderate to high-quality score (Additional file 1: Table S2).

### Triglycerides and the risk of cardiovascular diseases

**Fig. 1:**
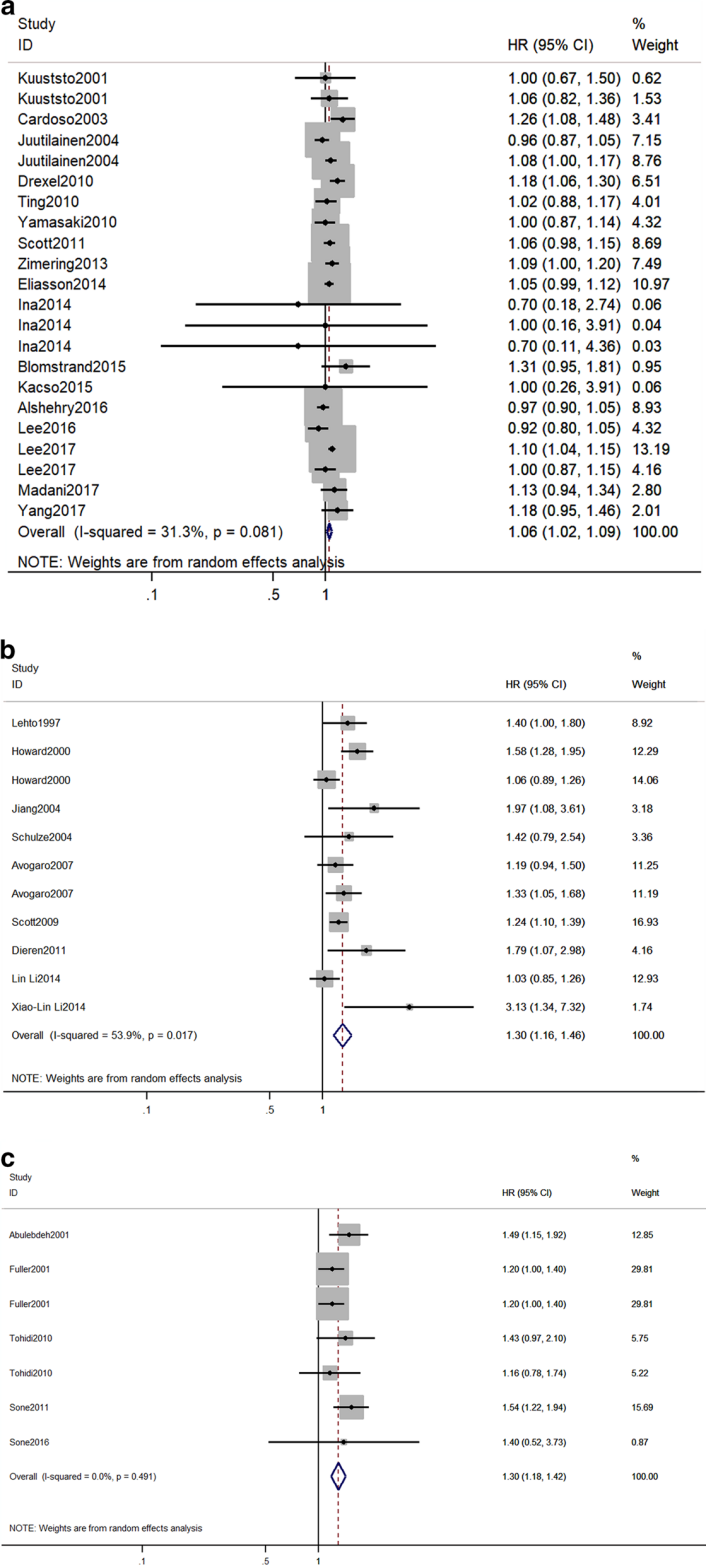
Forest plot for the effect of triglycerides on the risk of cardiovascular diseases in individuals with type 2 diabetes mellitus. RR and 95% CI for cardiovascular diseases and **a** 1 mmol/l increase in TG levels, **b** the highest versus the lowest category of TG, **c** 1 mmol/l increase in log TG levels. Studies are listed by date of publication. Boxes represent the RR and lines represent the 95% CI for individual studies. The diamonds and their width represent the pooled RRs and the 95% CI, respectively. *RR* relative risk

The effects of TG on the risk of CVD in T2DM are shown in Fig. 1. The random-effect model reveals that, the pooled maximum-adjusted RR (95% CI) associated with a 1-mmol/l increase in TG was 1.06 (1.02,1.09), I^2^ = 31.3%, p = 0.081) (Fig. 1a). A symmetric distribution in the funnel plot (Additional file 1: Figure S2) showed that there was no indication of publication bias, as the studies were evenly scattered on both sides of the average. Neither the Egger test (p = 0.83) or Begg’s test (p = 0.91) showed any publication bias. Comparing individuals in the highest category of TG to those in the lowest category of TG, the pooled maximum-adjusted RR (95% CI) for CVD was 1.30 (1.16,1.46), I^2^ = 53.9, p = 0.017 (Fig. 1b). The Egger test (p = 0.046) and Begg’s test (p = 0.043) showed an existing publication bias. The pooled maximum-adjusted RR (95% CI) for CVD in T2DM was 1.30 (1.18, 1.42) for each 1 mmol/l log TG increase, with no evidence of heterogeneity between the studies (I^2^ = 0.0%; p = 0.491) (Fig. 1c). Also, the Egger test (p = 0.455) and Begg test (p = 0.881) revealed no publication bias.

### Subgroup analysis

**Fig. 2:**
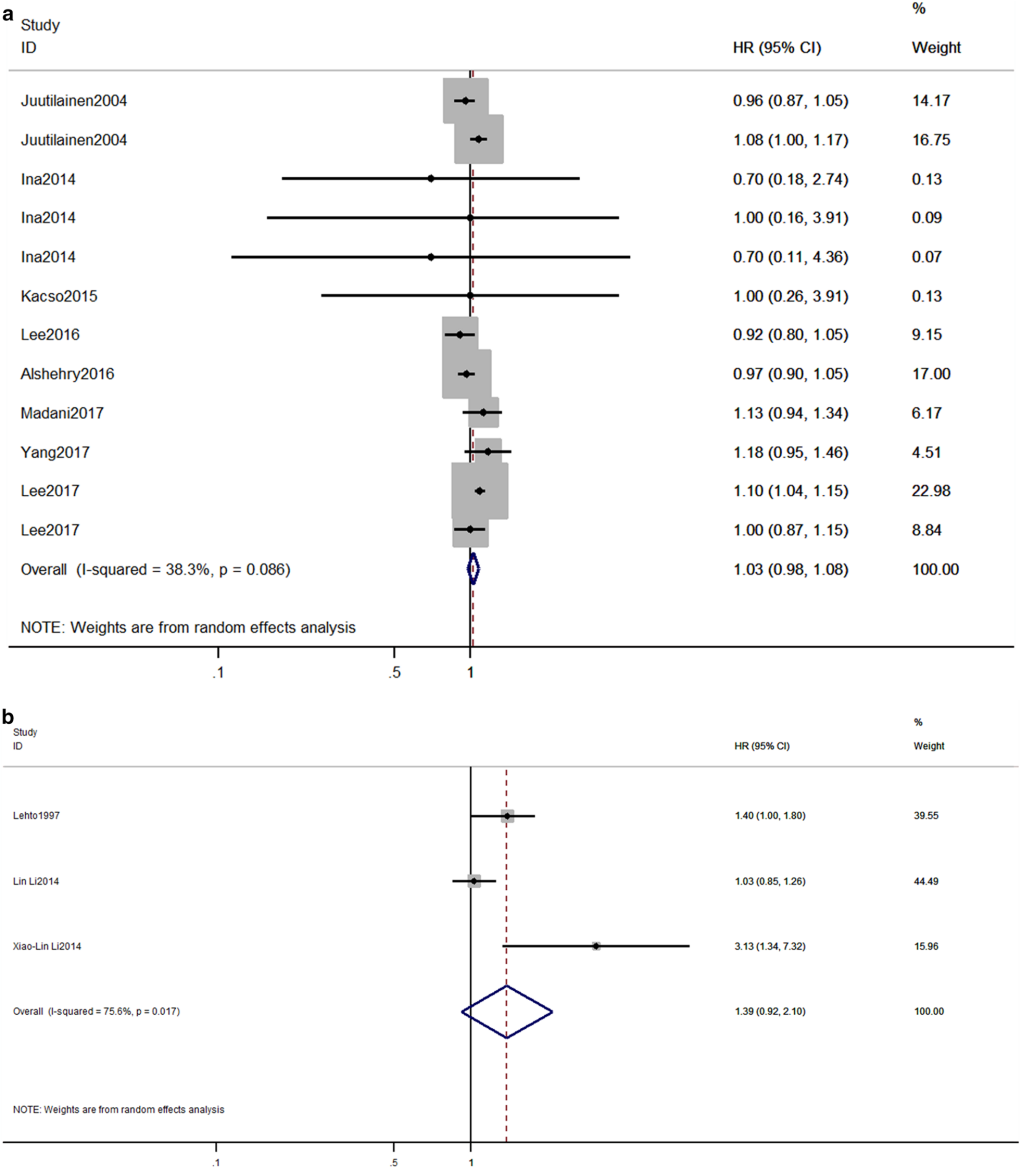
Forest plot for the effect of triglycerides on the risk of cardiovascular diseases in individuals with type 2 diabetes mellitus studies adjusted for other lipids parameters. RR and 95% CI for cardiovascular diseases and **a** 1 mmol/l increase in TG levels, **b** the highest versus the lowest category of TG. Studies are listed by date of publication. Boxes represent the RR and lines represent the 95% CI for individual studies. The diamonds and their width represent the pooled RRs and the 95% CI, respectively. *RR* relative risk

We performed subgroup meta-analyses, and the results are shown in Additional file 1: Table S3. The pooled estimates were derived from studies including eight studies with continuous TG and three studies with categories TG in patients with T2DM, the RR (95% CI) was adjusted for other lipids level, the calculated RR (95% CI) was 1.03 (0.98,1.08) (Fig. 2a) and 1.39 (0.92, 2.1) (Fig. 2b), respectively. The pooled RR (95% CI) of CVDs for TG categories, per 1 mmol/L TG increment and log TG were 1.31 (1.16, 1.47), 1.08 (1.04, 1.13) and 1.30 (1.18, 1.42), respectively for the studies without adjustment of other lipids level. It indicates that an increase in TG tend to associate with an increase in CVD risk but it might not be an independent risk factor for CVD in T2DM, further studies are warranted to elucidate such association. The associations of TG with CVD were consistent across an adjusted glycemic level and blood pressure in the subgroup. We also investigated the relationship between TG and the subgroup of CVD. In general, higher TG level tends to increase the risk of CHD but not a stroke.

When pooled estimates were derived independently for age ≥ 65 and < 65 years old recruited populations, TG tends to predict CVD risk in elderly patients with an age of < 65 years, but might not be a risk factor for CVD in T2DM patients with an age of ≥ 65 years. Analyses also indicated that the effects of elevated TG level on CVD risk were more pronounced among T2DM individuals with CVD history and particularly in the females, and showed more prominence in studies with longer follow-up durations. Besides, a stronger association was found in Asians compared to the Western populations. Finally, when pooled estimates were calculated for low and high (≥ 7%) baseline HbA1c, the baseline TG levels predicted CVD risk only in patients with HbA1c ≥ 7%. Moreover, when pooled estimates were calculated for renal function, the TG levels predicted the CVD events in patients with eGFR ≥ 60 min/ml/1.73 m^2^ and not with eGFR < 60 min/ml/1.73 m^2^.

## Discussion

In this systematic review and meta-analysis, we pooled the data for TG levels of 132, 044 T2DM subjects from 31 studies. To the best of our knowledge, this is the first meta-analysis to comprehensively investigated the predictive role of TG levels and assessed the factors influencing the predictive ability of TG. Our study findings provide useful implications for the development of disease prevention strategies. Firstly, the increased TG levels are associated with an increase in the risk of incident CVD. However, this association remained statistically insignificant when adjusted for other blood lipid parameters; it affirms the findings of previous studies reported that the TG is not an independent risk factor for incident CVD in the T2DM population [30, 31]. In contrast, the data from the general population confirmed that fasting serum TG are independently associated with the risk of CVD, even after adjustment for other lipid parameters [10, 32, 33]. Furthermore, our result indicated that a higher TG level tends to increase the risk of CVD mainly due to an increased risk of CHD but not a stroke. Secondly, the subgroup analyses on the impact of TG on CVD showed prominence among T2DM individuals with a mean age < 65 years. It is consistent with a recent cross-sectional study which found higher TG level was associated with an increased risk of CVD among the patients with short duration of diabetes, whereas, a lower TG level was associated with an increased risk of CVD in patients with longer duration of diabetes, and this association was mainly driven by CHD [34]. Older age tends to have a longer duration of diabetes in general. It had been reported that triglyceride-rich lipoproteins are the primary source of energetic heart lipids [35]. Moreover, it is well understood that elderly T2DM individuals have more severe insulin resistance and deteriorated Beta-cell function, resulting in poor glycemic control in patients with T2DM, which is a well‐established causal factor for CVD [36], and it is important to have a sufficient supply of TG to the heart because it is harder for the heart to use glucose as fuel. Therefore, a higher blood TG level might not be a risk factor for elderly T2DM patients but an essential fuel for their heart. Thirdly, a subgroup analysis indicated a weaker effects of elevated TG levels on CVD risk in male patients with T2DM compared to the female counterparts, and this is in line with Framingham Heart Study that reported higher TG to correlate more strongly with CVD risk in women than in men [37]. However, the reason behind this remains unknown, and future studies are invited to explore the pathogenic mechanism behind such inference. Moreover, in agreement with Liu’s meta-analysis in general population [38], a stronger association was also found in Asians compared with Western populations, and participants with the prior macrovascular disease compared with no history of macrovascular disease participants. Lastly, our study found that T2DM patient with eGFR ≤ 60 min/ml/1.73 m^2^ showed the blood TG as a protective factor against CVD, this finding coincides with the results of some observational studies based on hemodialysis patients [39, 40], according to their findings a higher cholesterol level was shown to predict a better survival. However, the exact reasons are not known and require further elucidation.

Evidence showed a TG involvement in atherogenesis indirectly [41, 42]. The progression of coronary atherosclerosis is powerfully stimulated by interactions between diabetes-associated factors and other factors such as abnormal lipid metabolism [43], and hypertriglyceridemia involved in these two important factors. Hypertriglyceridemia deteriorates diabetes by impairing the function of β-cell and causing peripheral insulin resistance (IR) [44]. TG overload in islets interferes with glucose metabolism, and the accumulation of metabolites derived from fatty-acid esterification impairs β-cell function [45–47]. The impairment of β-cell function decreases the glucose-induced insulin secretion, resulting in an increased glycemic level in T2DM patients, which significantly increases the risk of cardiovascular disease. Hypertriglyceridemia has a unidirectional relationship with peripheral IR. The metabolites of TG such as free fatty acids, diacylglycerol and others can regulate insulin-signaling pathways through activating several serine/threonine kinases, which suppress insulin receptor and tyrosine phosphorylation of insulin receptor substrates, inducing peripheral IR [48–50]. Many studies have indicated that IR leads to inflammation, altered coagulation and atherosclerosis [51–54]. Furthermore, an independent association between IR and CVD has been reported [55, 56]. TG involves in atherogenesis also by altering LDL-particle size. Some studies indicated that LDL-particle size showed a significantly negative correlation with the serum TG levels [57, 58], this means that when serum TG is elevated, the LDL-particle size became smaller. The Québec Cardiovascular Study demonstrated that patients had LDL-particle size of 25.5 nm or smaller, the CHD incidence increased significantly as the serum LDL-C level increased, while in patients having large LDL-particle sizes of 26.0 nm or greater, no significant difference in CAD events was observed according to the absolute serum LDL-C level [59]. Smaller LDL-particle size showed a powerful atherogenic effect [41]. Moreover, TG involved in atherogenesis by other mechanisms. TG metabolites, i.e., chylomicrons, very low-density lipoprotein, and remnant-like particle cholesterol, which are TG-rich lipoproteins, and, apolipoprotein (apo) C-II, and apo C-III which are involved in the metabolic process, etc., have been demonstrated to be involved in the progression of atherosclerosis [41]. In addition, mounting evidence suggested that TG may stimulate atherogenesis through the production of proinflammatory cytokines, fibrinogen and coagulation factors and impairment of fibrinolysis [60, 61].

However, a series of experimental studies based on alloxan-diabetic rabbits demonstrated that the lipoproteins in the hypertriglyceridemic diabetic rabbits are much larger than in normotriglyceridemic rabbits, and the plasma lipoproteins that contain most of the plasma cholesterol are so large in size (the diameter larger than 75 nm) that they are not able to enter the arterial wall. A reduced aortic cholesterol influx means that the subendothelial macrophages and smooth muscle cells are exposed to relatively small amounts of cholesterol, this probably explains the slow development of atherosclerotic plaques in severely hypertriglyceridemic diabetic rabbits [62–64]. However, these findings did not mean that the very large triglyceride-rich lipoproteins in diabetes are harmless, it means these are less atherogenic than smaller lipoproteins and may partially explain that TG is not an independent risk factor for CVD in diabetes patients.

Even in diabetes patients receiving appropriate treatment with statins, a substantial residual cardiovascular risk remains [65–67]. Also, the American Diabetes Association (ADA) recommending TG-lowering as an important secondary target in patients with diabetes [68]. There are different triglyceride-lowering drugs available. Clinically, the drugs that predominantly lower the triglyceride levels are fibrates, fish oils and nicotinic acid. The statins, ezetimibe, proprotein convertase subtilisin/kexin type 9 inhibitors are LDL-cholesterol lowering agents which also reduces the triglycerides level. Besides, there are newer triglyceride-lowering agents currently under evaluation. These novel specific agents for high TG include newer fibrates such as pemafibrate; newer omega-3 FAs such as eicosapentaenoic acid; combined peroxisome proliferator-activated receptor alpha/gamma agonists such as aleglitazar; and new drug classes of apolipoprotein CIII antisense therapies, microsomal triglyceride transfer protein inhibition such as lomitapide, and diacylglycerol acyltransferase inhibitors such as pradigastat; and anti-angiopoietin-like protein 3 or anti-angiopoietin-like protein 4. Furthermore, probiotics and gut microbiome is a new therapeutic modulation for hypertriglyceridemia. Many new TG reducing therapies are in clinical trials. In addition to the REDUCE-IT study, the Outcomes Study to Assess Statin Residual Risk Reduction With EpaNova in High CV Risk Patients With Hypertriglyceridemia (STRENGTH) and the Pemafibrate to Reduce Cardiovascular Outcomes by Reducing Triglycerides In Patients With Diabetes (PROMINENT) studies are ongoing large CV outcomes trials involving high-risk CVD patients, including a large percentage of patients with diabetes, undergoing statin therapy [17, 69–72].

We acknowledge that our study had several limitations. First, most of the included studies were observational studies, but several were randomized control trials, which might have led to the heterogeneities. Furthermore, because our study is a literature-based meta-analysis, the lack of access to individual patient data might lead to the heterogeneities in TG contrast level and adjustments. Meanwhile, the differences in the definition of CVD, effect size types (OR, RR, or HR) have to be mentioned as well. However, a random-effects model and subgroup analyses were conducted to minimize the impact of heterogeneity. Second, a single baseline TG estimation may have led to the misclassification of study participants in each category and multiple determination of TG during follow up would increase the precision. Third, although we used the most fully adjusted RR from each study to calculate the pooled result, there are so many confounders that may have an effect on the outcome, such as lifestyle and socio‐economic status, e.g., cigarettes smoking and alcohol drinking, antihypertension and lipid‐lowering drugs and antidiabetic medicine. Further well designed clinical trials are needed to overcome all these confounders. Finally, evidence suggests that postprandial (non-fasting) TG levels may have a stronger association with CVD than fasting levels [73] in the general population, but in our study, only one study had reported on the relationship between postprandial TG and CVD, so we could not evaluate this issue very robustly.

## Conclusion

Findings from this meta-analysis of prospective studies indicate that an increased TG level tends to associate with an increase in the risk of incident CVD in T2DM. The results of our study and the available evidence, do not downgrade the importance of TG control as cornerstones for the prevention and the management of CVD in T2DM. Further, well designed community-based cohort studies or interventional studies are warranted to confirm TG as a crucial risk factor for CVD.

## Additional file

**Additional file 1: Figure S1.** Flow diagram of study selection. **Figure S2.** Publication bias for cardiovascular diseases risk per 1 mmol/l triglycerides level increase in type 2 diabetes. **Table S1.** Characteristics of studies of triglycerides and cardiovascular diseases in individuals with type 2 diabetes mellitus. **Table S2.** Quality assessments—Newcastle–Ottawa quality assessment scale. **Table S3.** Subgroup analyses of triglycerides level with cardiovascular diseases risk in individuals with type 2 diabetes mellitus.

## Abbreviations

TG: triglycerides
CVD: cardiovascular diseases
CHD: coronary heart diseases
T2DM: type 2 diabetes mellitus
RR: risk ratios
CI: confidence intervals
log: logarithm
WHO: World Health Organization
LDL-C: low-density lipoprotein cholesterol
MOOSE: Meta-Analysis of Observational Studies in Epidemiology
CNKI: China National Knowledge Infrastructure
Mesh: medical subject heading
HR: hazard ratio
NOS: New Castle–Ottawa Scale
HbA1c: glycated hemoglobin
eGFR: estimated glomerular filtration rate
HDL-C: high-density lipoprotein cholesterol
IR: insulin resistance
apo: apolipoprotein
ADA: American Diabetes Association
