# Supplementary material for: Serum triglycerides as a risk factor for cardiovascular diseases in type 2 diabetes mellitus: a systematic review and meta-analysis of prospective studies
Source: Cardiovasc Diabetol. 2019 Apr 15;18:48. doi: 10.1186/s12933-019-0851-z (PMC6466658; doi:10.1186/s12933-019-0851-z)
Supplement: Supplementary file 1 — Additional file 1: Figure S1. Flow diagram of study selection. Figure S2. Publication bias for cardiovascular diseases risk per 1 mmol/l triglycerides level increase in type 2 diabetes. Table S1. Characteristics of studies of triglycerides and cardiovascular diseases in individuals with type 2 diabetes mellitus. Table S2. Quality assessments—Newcastle–Ottawa quality assessment scale. Table S3. Subgroup analyses of triglycerides level with cardiovascular diseases risk in individuals with type 2 diabetes mellitus. [file 12933_2019_851_MOESM1_ESM.doc]

**Additional file 1: Figure S1.** Flow diagram of study selection.

Records identified through database searching(n=5282)

PUBMED:1165

EMBASE:884

COCHRANE:840

Clinicaltrails.gov:372

Web Of Science and Others:2021

Full text articles assessed for eligibility(n=301)

Records screened by title and abstract(n=3455)

Duplicate records removed(n=1508)

Reviews and meta-analysis removed(n=319)

Irrelevant records removed(n=3154)

Records excluded(n=271)

n=34 studies do not have relevant outcome data

n=38 studies without a longitudinal design

n=150 studies not using TG as predictor variable

n=10 duplicate reports of the same study

n=2 studies do not calculate 95%CI

n=37 studies full-text not available

Studies included in meta-analysis(n=31)

1 additional article identified from manual search of reference lists

**Additional file 1: Figure S2.** Publication bias for cardiovascular diseases risk per 1 mmol/l triglycerides level increase in type 2 diabetes. Circles represent individual studies of the meta-analysis and the vertical line the pooled estimate of the relative risk for cardiovascular diseases.

Additional file 1: Table S1. Characteristics of studies of Triglycerides and Cardiovascular Diseases in individuals with type 2 diabetes mellitus

| Study ID | Country | Sample Size at Baseline, n | Mean Follow-up Time, y | Mean Age at Baseline, y | Male (%) | Number of events | TG variable | Maximum adjustments ^b^ |
| --- | --- | --- | --- | --- | --- | --- | --- | --- |
| Lehto1997 [1] | Finland | 1059 | 7.2 | 58.2 | 54.86 | 256 | category TG | age, sex, area, previous MI, TC, FBG, and diabetes duration. |
| Howard2000 [2] | American | 2034 | 4.8 | 57.28 | 35 | 521 | category TG | sex, age and center |
| Abulebdeh2001 [3] | American | 449 | 13 | 57 | 47 | 331 | log TG | age, glucose, smoking |
| Kuuststo2001 [4] | Finland | 229 | 7 | 69.37 | 32.3 | 70 | continuous TG | sex |
| Fuller2001 [5] | Britain | 3483 | 12 | 46.84 | 47.69 | 591 | log TG | sex, age |
| Cardoso2003 [6] | Brazil | 471 | 4.75 | 61^a^ | 34.2 | 106 | continuous TG | gender, age, FBG, cerebral or peripheral vascular disease, cardiac disease, diabetes duration, neuropathy |
| Jiang2004 [7] | American | 746 | 6 | 63.1 | 100 | 103 | category TG | age, BMI, family history of MI, physical activity, smoking, alcohol, fasting status, hypertension, aspirin use, and HbA1c. |
| Juutilainen2004 [8] | Finland | 835 | 13 | 57.8 | 51.4 | 277 | continuous TG | sex, age and area of residence, and variables enforced into the model recurrent smoking, BMI, SBP, TC, HDL-C, FBG, diabetes duration |
| Schulze2004 [9] | American | 921 | 7.4 | 58.3 | 0 | 122 | category TG | age, physical activity, alcohol intake, parental history of CHD, hypertension, aspirin use, smoking, postmenopausal hormone use and BMI |
| Avogaro2007 [10] | Italy | 11644 | 4 | 65.2 | 48.2 | 881 | category TG | sex |
| Scott2009 [11] | New Zealand | 9795 | 5 | 62.2 | 63 | 683 | category TG | sex, age, prior CVD, and baseline HbA1c |
| Drexel2010 [12] | Austria | 116 | 5.6 | 63 | 69 | 52 | continuous TG | age, gender, BMI, smoking, and hypertension |
| Ting2010 [13] | Hong Kong, China | 4521 | 4.9 | 54.8^a^ | 46.3 | 371 | continuous TG | age, smoking status, duration of diabetes, HbA1c, use of statins, fibrates, gliclazide and rosiglitazone during follow-up, years of enrolment |
| Yamasaki2010 [14] | Japan | 485 | 3 | 66.8 | 57.7 | 57 | continuous TG |  |
| Tohidi2010 [15] | Iran | 1021 | 8.6 | 54.8 | 40.5 | 189 | log TG | sex, age, family history of premature CVD, SBP and aspirin use, DBP, smoking, antihypertensive drug |
| Scott2011 [16] | New Zealand | 4900 | 5 | 62.2 | 63 |  | continuous TG | age, sex, prior CVD, baseline HbA1c and creatinine. |
| Dieren2011 [17] | Netherlands | 1337 | 8 |  |  | 116 | category TG | age, sex, cohort, smoking, SBP, BMI, alcohol, HbA1c level, diabetes duration, energy intake, diabetes medication, physical activity and postprandial time |
| Sone2011 [18] | Japan | 1771 | 7.86 | 58.4 | 53.08 | 115 | log TG | sex, age, diabetes duration, BMI, SBP, HbA1c, smoking, and alcohol |
| Zimering2013 [19] | American | 399 | 7.5 | 59.3 | 96.5 | 105 | continuous TG | prior CVD, age, diabetes duration, baseline fibroblast growth factor, intensive treatment. |
| Ina2014 [20] | Japan | 462 | 2 | 67.4 | 49.7 | 19 | continuous TG | age, sex, SBP, DBP, HbA1c, plasma lipid levels and antihyperlipidemic agents |
| Lin Li2014 [21] | Britain | 21,998 | 9 | 57 | 54.4 | 895 | category TG | age, gender, BMI, smoking, alcohol, hypertension, peripheral vascular disease, current diabetes treatment, diabetes duration, cardiovascular medications, baseline lipid-modifying therapy, LDL-C, HDL-C, HbA1c. |
| Xiao-Lin Li2014 [22] | China | 329 | 1 | 59.3 | 73.3 | 47 | category TG | age, gender, BMI, traditional cardiovascular risk factors, other lipid parameters and hematological index of differential at baseline |
| Eliasson2014 [23] | Sweden | 46,786 | 5.8 | 59.8 | 60.3 | 2590 | continuous TG | lipid-lowering drugs, age, sex, duration, type of hypoglycemic treatment, HbA1c, smoking, BMI, albuminuria >20mg/min, SBP, antihypertensive drugs, history of CVD or heart failure, atrial fibrillation |
| Blomstrand2015 [24] | Sweden | 406 | 5.58 | 60.7 | 68 | 19 | continuous TG |  |
| Kacso2015 [25] | Romania | 77 | 3 | 61.08 | 62.33 | 36 | continuous TG | glycemia, HDL-C, UA, albumin, calcium, transferrin, ferritin, statins. |
| Lee2016 [26] | Republic of Korea | 1136 | 3.67 | 61.7 | 65.6 | 191 | continuous TG | diabetes duration, age, sex, BMI, hypertension, CVD history, log ACR, eGFR, HDL-C, LDL-C, HbA1c. |
| Alshehry2016 [27] | Australia | 3779 | 5 | 67^a^ | 61.1 | 698 | continuous TG | age, sex, BMI, SBP, HbA1c, HDL-C, eGFR, diabetes duration, CRP, history of CVD and heart failure, use of antihypertensive medication, use of antiplatelet medication, and exercise. |
| Sone2016 [28] | Japan | 668 | 5.3 | 58.66 | 44.2 | 70 | log TG |  |
| Madani2017 [29] | Iran | 481 | 5~8 | 54.9 | 43.37 | 69 | continuous TG | BMI, HbA1c, FBG, PPG, TG, creatinine, gender, age and insulin user. |
| Yang2017 [30] | China | 1,447 | 1.69 | 59.2 | 70.5 | 96 | continuous TG | age, sex, BMI, left ventricle ejection fraction, current smoking, previous PCI/CABG and TC; HbA1c; CRP, SBP, DBP and fibrinogen. |
| Lee2017 [31] | Taiwan, China | 8259 | 6.29 | 62 | 52 | 1057 | continuous TG | age, sex, hypertension, diabetic retinopathy and neuropathy, HbA1c SD, mean HbA1c, HDL-C, eGFR, ACEI and/or ARB use, aspirin use, statin and/or fibrate use, and insulin use |

^a^: Median

^b^: Abbreviations: BMI: body mass index; SBP: systolic blood pressure; DBP: diastolic blood pressure; HbA1c: glycated hemoglobin; FBG: fasting blood glucose; PPG: postprandial blood glucose; TC: total cholesterol; CVD: cardiovascular diseases; eGFR: estimated glomerular filtration rate; HDL-C: high-density lipoprotein cholesterol; LDL-C: low-density lipoprotein cholesterol; PCI: percutaneous coronary intervention; CABG: coronary artery bypass grafting; CRP:C-reactive protein; ACEI: angiotensin converting enzyme inhibitor; ARB: angiotensin receptor blocker; UA: uric acid; MI: myocardial infarction.

Additional file 1: Table S2. Quality assessments—Newcastle-Ottawa quality assessment scale

| Study ID | 01.representativeness of the exposed cohort | 02.selection of the unexposed cohort | 03.ascertainment of exposure | 04.outcome of interest not present at start of study | 05.control for important factor or additional factor | 06.outcome assessment | 07.follow-up long enough for outcomes to occur | 08.adequacy of follow-up of cohort | total |
| --- | --- | --- | --- | --- | --- | --- | --- | --- | --- |
| Lehto1997 | ★ | ★ | ★ |  | ★★ | ★ | ★ | ★ | 8 |
| Howard2000 |  | ★ | ★ | ★ | ★★ | ★ | ★ | ★ | 8 |
| Abulebdeh2001 | ★ | ★ | ★ | ★ | ★★ | ★ | ★ | ★ | 9 |
| Kuuststo2001 | ★ | ★ | ★ |  |  | ★ | ★ | ★ | 6 |
| Fuller2001 | ★ | ★ | ★ |  | ★ | ★ | ★ | ★ | 7 |
| Cardoso2003 | ★ | ★ | ★ |  | ★★ | ★ | ★ | ★ | 8 |
| Jiang2004 |  | ★ | ★ | ★ | ★★ | ★ | ★ | ★ | 8 |
| Juutilainen2004 | ★ | ★ | ★ | ★ | ★★ | ★ | ★ | ★ | 9 |
| Schulze2004 |  | ★ | ★ | ★ | ★★ | ★ | ★ | ★ | 8 |
| Avogaro2007 |  | ★ | ★ | ★ |  | ★ | ★ | ★ | 6 |
| Scott2009 |  | ★ | ★ |  | ★★ | ★ | ★ | ★ | 7 |
| Drexel2010 |  | ★ | ★ |  | ★★ | ★ | ★ | ★ | 7 |
| Ting2010 |  | ★ | ★ | ★ | ★★ | ★ | ★ | ★ | 8 |
| Yamasaki2010 |  | ★ | ★ | ★ |  | ★ | ★ | ★ | 6 |
| Tohidi2010 | ★ | ★ | ★ | ★ | ★★ |  | ★ | ★ | 8 |
| Scott2011 |  | ★ | ★ |  | ★★ | ★ | ★ | ★ | 7 |
| Dieren2011 | ★ | ★ | ★ | ★ | ★★ | ★ | ★ | ★ | 9 |
| Sone2011 | ★ | ★ | ★ | ★ | ★★ | ★ | ★ |  | 8 |
| Zimering2013 |  | ★ | ★ |  | ★★ | ★ | ★ | ★ | 7 |
| Ina2014 |  | ★ | ★ | ★ | ★★ | ★ |  | ★ | 7 |
| Lin Li2014 |  | ★ | ★ | ★ | ★★ | ★ | ★ | ★ | 8 |
| Xiao-Lin Li2014 |  | ★ | ★ |  | ★★ |  |  | ★ | 5 |
| Eliasson2014 | ★ | ★ | ★ |  | ★★ | ★ | ★ | ★ | 8 |
| Blomstrand2015 |  | ★ | ★ |  |  | ★ | ★ |  | 4 |
| Kacso2015 |  | ★ | ★ |  | ★★ | ★ | ★ | ★ | 7 |
| Lee2016 |  | ★ | ★ |  | ★★ | ★ | ★ | ★ | 7 |
| Alshehry2016 |  | ★ | ★ |  | ★★ | ★ | ★ | ★ | 7 |
| Sone2016 |  | ★ | ★ | ★ | ★★ | ★ | ★ |  | 7 |
| Madani2017 |  | ★ | ★ | ★ | ★★ | ★ | ★ | ★ | 8 |
| Yang2017 |  | ★ | ★ |  | ★★ | ★ |  | ★ | 6 |
| Lee2017 | ★ | ★ | ★ |  | ★★ | ★ | ★ | ★ | 8 |

01 Selection: No ★ is given selected group e.g. only man or woman group;

05 Comparability: one ★ is given if adjusted for age and sex, two ★ are given if adjusted for age, sex and others.

07 Follow-up length: No ★ is given if < 3 years;

08 Follow-up adequacy: No ★ is given if number of lost > 20% or without description of those lost.

Additional file 1: Table S3. Subgroup analyses of triglycerides level with cardiovascular diseases risk in individuals with type 2 diabetes mellitus

|  | continuous analyses | | | | categorical analyses | | | | logarithm analyses | | | |
| --- | --- | --- | --- | --- | --- | --- | --- | --- | --- | --- | --- | --- |
| Subgroups | Studies, n | RR (95 % CI) | Heterogeneity | | Studies, n | RR (95 % CI) | Heterogeneity | | Studies, n | RR (95 % CI) | Heterogeneity | |
|  |  |  | P | I², % |  |  | P | I², % |  |  | P | I², % |
| adjusted for other lipids level | | | | | | | | | | | | |
| Yes | 8 | 1.03(0.98,1.08) | 0.086 | 38.3 | 3 | 1.39(0.92,2.10) | 0.017 | 75.6 | 0 |  |  |  |
| No | 9 | 1.08(1.04,1.13) | 0.287 | 17 | 6 | 1.31(1.16,1.47) | 0.077 | 45.3 | 5 | 1.30(1.18,1.42) | 0.491 | 0 |
| adjusted for glycemia | | | | | | | | | | | | |
| Yes | 13 | 1.05(1.01,1.09) | 0.069 | 36.1 | 6 | 1.37(1.12,1.67) | 0.025 | 61.1 | 2 | 1.52(1.28,1.80) | 0.852 | 0 |
| No | 4 | 1.10(1.04,1.16) | 0.424 | 0 | 3 | 1.28(1.09,1.50) | 0.066 | 54.7 | 3 | 1.22(1.09,1.36) | 0.931 | 0 |
| adjusted for blood pressure | | | | | | | | | | | | |
| Yes | 8 | 1.04(1.00,1.09) | 0.024 | 50 | 5 | 1.59(1.08,2.35) | 0.015 | 67.8 | 2 | 1.43(1.20,1.71) | 0.495 | 0 |
| No | 9 | 1.08(1.03,1.13) | 0.488 | 0 | 4 | 1.27(1.14,1.42) | 0.1 | 45.8 | 3 | 1.25(1.12,1.39) | 0.512 | 0 |
| subgroup of CVD | | | | | | | | | | | | |
| CHD | 4 | 1.06(0.98,1.14) | 0.085 | 51.0 | 6 | 1.60(1.29,1.98) | 0.03 | 54.8 | 2 | 1.29(1.13,1.48) | 0.218 | 32.4 |
| stroke | 0 |  |  |  | 3 | 1.39(0.88,2.19) | 0.03 | 71.4 | 2 | 1.15(1.00,1.33) | 0.87 | 0 |
| baseline age | | | | | | | | | | | | |
| <65 years old | 14 | 1.07(1.04,1.11) | 0.065 | 38.3 | 9 | 1.28(1.12,1.46) | 0.013 | 58.8 | 5 | 1.30(1.18,1.42) | 0.491 | 0 |
| ≥65 years old | 5 | 0.99(0.93,1.04) | 0.994 | 0 | 1 | 1.33(1.05,1.68) |  |  | 0 |  |  |  |
| gender | | | | | | | | | | | | |
| male | 2 | 0.96(0.88,1.05) | 0.831 | 0 | 3 | 1.19(0.95,1.49) | 0.138 | 49.5 | 2 | 1.23(1.06,1.44) | 0.413 | 0 |
| female | 2 | 1.08(1.00,1.16) | 0.883 | 0 | 3 | 1.46(1.26,1.70) | 0.561 | 0 | 2 | 1.19(1.02,1.39) | 0.888 | 0 |
| mix | 15 | 1.06(1.03,1.10) | 0.078 | 34.2 | 5 | 1.32(1.08,1.61) | 0.031 | 62.3 | 3 | 1.51(1.28,1.79) | 0.97 | 0 |
| duration of follow-up | | | | | | | | | | | | |
| <5 years | 7 | 1.05(0.96,1.15) | 0.183 | 29.5 | 3 | 1.33(1.08,1.63) | 0.011 | 69.4 | 0 |  |  |  |
| ≥5 years | 10 | 1.06(1.02,1.10) | 0.092 | 36.4 | 6 | 1.28(1.10,1.50) | 0.13 | 41.2 | 5 | 1.30(1.18,1.42) | 0.491 | 0 |
| geographic location | | | | | | | | | | | | |
| Europe/America | 7 | 1.05(1.02,1.09) | 0.577 | 0 | 8 | 1.27(1.14,1.42) | 0.046 | 47.6 | 2 | 1.25(1.11,1.40) | 0.324 | 11.3 |
| Asia-Pacific | 10 | 1.06(1.00,1.12) | 0.022 | 49.4 | 1 | 3.13(1.34,7.32) |  |  | 3 | 1.43(1.20,1.71) | 0.703 | 0 |
| free CVD history at baseline | | | | | | | | | | | | |
| Yes | 6 | 1.04(0.98,1.10) | 0.346 | 10.7 | 4 | 1.31(1.12,1.54) | 0.06 | 52.8 | 4 | 1.45(1.26,1.68) | 0.832 | 0 |
| No | 12 | 1.07(1.02,1.11) | 0.034 | 45.1 | 5 | 1.32(1.07,1.63) | 0.028 | 63.2 | 1 | 1.20(1.07,1.35) |  |  |
| baseline HbA1c (%) | | | | | | | | | | | | |
| <7 | 3 | 1.07(0.99,1.16) | 0.458 | 0 | 3 | 1.51(0.99,2.32) | 0.098 | 56.9 | 0 |  |  |  |
| ≥7 | 14 | 1.05(1.02,1.09) | 0.05 | 37.7 | 4 | 1.24(1.07,1.45) | 0.155 | 40 | 3 | 1.43(1.20,1.71) | 0.703 | 0 |
| renal function | | | | | | | | | | | | |
| eGFR≥60min/ml/1.73m² | 15 | 1.07(1.03,1.10) | 0.122 | 28.3 |  |  |  |  |  |  |  |  |
| eGFR<60min/ml/1.73m² | 3 | 0.96(0.87,1.05) | 0.668 | 0 |  |  |  |  |  |  |  |  |
| categorical | | | | | | | | | | | | |
| categorical two |  |  |  |  | 3 | 1.26(1.15,1.38) | 0.802 | 0 |  |  |  |  |
| categorical three/four |  |  |  |  | 6 | 1.41(1.12,1.78) | 0.002 | 70.9 |  |  |  |  |

RR: relative risk; CI: confidence interval; CVD: cardiovascular diseases; HbA1c: glycated hemoglobin; eGFR: estimated glomerular filtration rate.

Reference

1. Lehto S, Ronnemaa T, Haffner SM, Pyorala K, Kallio V, Laakso M: Dyslipidemia and hyperglycemia predict coronary heart disease events in middle-aged patients with NIDDM. Diabetes 1997, 46(8):1354-1359.

2. Howard BV, Robbins DC, Sievers ML, Lee ET, Rhoades D, Devereux RB, Cowan LD, Gray RS, Welty TK, Go OT et al: LDL cholesterol as a strong predictor of coronary heart disease in diabetic individuals with insulin resistance and low LDL: The Strong Heart Study. Arteriosclerosis, thrombosis, and vascular biology 2000, 20(3):830-835.

3. Abu-Lebdeh HS, Hodge DO, Nguyen TT: Predictors of macrovascular disease in patients with type 2 diabetes mellitus. Mayo Clin Proc 2001, 76(7):707-712.

4. Kuusisto J, Lempiainen P, Mykkanen L, Laakso M: Insulin resistance syndrome predicts coronary heart disease events in elderly type 2 diabetic men. Diabetes care 2001, 24(9):1629-1633.

5. Fuller JH, Stevens LK, Wang SL: Risk factors for cardiovascular mortality and morbidity: the WHO Mutinational Study of Vascular Disease in Diabetes. Diabetologia 2001, 44 Suppl 2:S54-64.

6. Cardoso CR, Salles GF, Deccache W: Prognostic value of QT interval parameters in type 2 diabetes mellitus: results of a long-term follow-up prospective study. Journal of diabetes and its complications 2003, 17(4):169-178.

7. Jiang R, Schulze MB, Li T, Rifai N, Stampfer MJ, Rimm EB, Hu FB: Non-HDL cholesterol and apolipoprotein B predict cardiovascular disease events among men with type 2 diabetes. Diabetes care 2004, 27(8):1991-1997.

8. Juutilainen A, Kortelainen S, Lehto S, Ronnemaa T, Pyorala K, Laakso M: Gender difference in the impact of type 2 diabetes on coronary heart disease risk. Diabetes care 2004, 27(12):2898-2904.

9. Schulze MB, Shai I, Manson JE, Li T, Rifai N, Jiang R, Hu FB: Joint role of non-HDL cholesterol and glycated haemoglobin in predicting future coronary heart disease events among women with type 2 diabetes. Diabetologia 2004, 47(12):2129-2136.

10. Avogaro A, Giorda C, Maggini M, Mannucci E, Raschetti R, Lombardo F, Spila-Alegiani S, Turco S, Velussi M, Ferrannini E et al: Incidence of coronary heart disease in type 2 diabetic men and women: impact of microvascular complications, treatment, and geographic location. Diabetes care 2007, 30(5):1241-1247.

11. Scott R, O'Brien R, Fulcher G, Pardy C, D'Emden M, Tse D, Taskinen MR, Ehnholm C, Keech A, Fenofibrate I et al: Effects of fenofibrate treatment on cardiovascular disease risk in 9,795 individuals with type 2 diabetes and various components of the metabolic syndrome: the Fenofibrate Intervention and Event Lowering in Diabetes (FIELD) study. Diabetes care 2009, 32(3):493-498.

12. Drexel H, Aczel S, Marte T, Vonbank A, Saely CH: Factors predicting cardiovascular events in statin-treated diabetic and non-diabetic patients with coronary atherosclerosis. Atherosclerosis 2010, 208(2):484-489.

13. Ting RZ, Yang X, Yu LW, Luk AO, Kong AP, Tong PC, So WY, Chan JC, Ma RC: Lipid control and use of lipid-regulating drugs for prevention of cardiovascular events in Chinese type 2 diabetic patients: a prospective cohort study. Cardiovascular diabetology 2010, 9:77.

14. Yamasaki Y, Nakajima K, Kusuoka H, Izumi T, Kashiwagi A, Kawamori R, Shimamoto K, Yamada N, Nishimura T: Prognostic value of gated myocardial perfusion imaging for asymptomatic patients with type 2 diabetes: the J-ACCESS 2 investigation. Diabetes care 2010, 33(11):2320-2326.

15. Tohidi M, Hatami M, Hadaegh F, Safarkhani M, Harati H, Azizi F: Lipid measures for prediction of incident cardiovascular disease in diabetic and non-diabetic adults: results of the 8.6 years follow-up of a population based cohort study. Lipids in health and disease 2010, 9:6.

16. Scott R, Donoghoe M, Watts GF, O'Brien R, Pardy C, Taskinen MR, Davis TM, Colman PG, Manning P, Fulcher G et al: Impact of metabolic syndrome and its components on cardiovascular disease event rates in 4900 patients with type 2 diabetes assigned to placebo in the FIELD randomised trial. Cardiovascular diabetology 2011, 10:102.

17. van Dieren S, Nothlings U, van der Schouw YT, Spijkerman AM, Rutten GE, van der AD, Sluik D, Weikert C, Joost HG, Boeing H et al: Non-fasting lipids and risk of cardiovascular disease in patients with diabetes mellitus. Diabetologia 2011, 54(1):73-77.

18. Sone H, Tanaka S, Tanaka S, Iimuro S, Ishibashi S, Oikawa S, Shimano H, Katayama S, Ohashi Y, Akanuma Y et al: Comparison of various lipid variables as predictors of coronary heart disease in Japanese men and women with type 2 diabetes: subanalysis of the Japan Diabetes Complications Study. Diabetes care 2012, 35(5):1150-1157.

19. Zimering MB, Anderson RJ, Ge L, Moritz TE, Duckworth WC, Investigators for the V: Basic fibroblast growth factor predicts cardiovascular disease occurrence in participants from the veterans affairs diabetes trial. Frontiers in endocrinology 2013, 4:183.

20. Ina K, Hayashi T, Araki A, Kawashima S, Sone H, Watanabe H, Ohrui T, Yokote K, Takemoto M, Kubota K et al: Importance of high-density lipoprotein cholesterol levels in elderly diabetic individuals with type IIb dyslipidemia: A 2-year survey of cardiovascular events. Geriatr Gerontol Int 2014, 14(4):806-810.

21. Li L, Ambegaonkar BM, Reckless JP, Jick S: Association of a reduction in low-density lipoprotein cholesterol with incident cardiovascular and cerebrovascular events among people with type 2 diabetes mellitus. European journal of preventive cardiology 2014, 21(7):855-865.

22. Li XL, Hong LF, Luo SH, Guo YL, Zhu CG, Sun J, Dong Q, Qing P, Xu RX, Liu J et al: Impact of admission triglyceride for early outcome in diabetic patients with stable coronary artery disease. Lipids in health and disease 2014, 13:73.

23. Eliasson B, Gudbjornsdottir S, Zethelius B, Eeg-Olofsson K, Cederholm J, National Diabetes R: LDL-cholesterol versus non-HDL-to-HDL-cholesterol ratio and risk for coronary heart disease in type 2 diabetes. European journal of preventive cardiology 2014, 21(11):1420-1428.

24. Blomstrand P, Engvall M, Festin K, Lindstrom T, Lanne T, Maret E, Nystrom FH, Maret-Ouda J, Ostgren CJ, Engvall J: Left ventricular diastolic function, assessed by echocardiography and tissue Doppler imaging, is a strong predictor of cardiovascular events, superior to global left ventricular longitudinal strain, in patients with type 2 diabetes. Eur Heart J Cardiovasc Imaging 2015, 16(9):1000-1007.

25. Kacso IM, Potra AR, Bondor CI, Moldovan D, Rusu C, Patiu IM, Racasan S, Orasan R, Vladutiu D, Spanu C et al: Adiponectin predicts cardiovascular events in diabetes dialysis patients. Clin Biochem 2015, 48(13-14):860-865.

26. Lee E, Oh HJ, Park JT, Han SH, Ryu DR, Kang SW, Yoo TH: The Incidence of Cardiovascular Events Is Comparable Between Normoalbuminuric and Albuminuric Diabetic Patients With Chronic Kidney Disease. Medicine 2016, 95(15):e3175.

27. Alshehry ZH, Mundra PA, Barlow CK, Mellett NA, Wong G, McConville MJ, Simes J, Tonkin AM, Sullivan DR, Barnes EH et al: Plasma Lipidomic Profiles Improve on Traditional Risk Factors for the Prediction of Cardiovascular Events in Type 2 Diabetes Mellitus. Circulation 2016, 134(21):1637-1650.

28. Sone H, Nakagami T, Nishimura R, Tajima N, Group MS: Comparison of lipid parameters to predict cardiovascular events in Japanese mild-to-moderate hypercholesterolemic patients with and without type 2 diabetes: Subanalysis of the MEGA study. Diabetes research and clinical practice 2016, 113:14-22.

29. Hashemi Madani N, Ismail-Beigi F, Khamseh ME, Malek M, Ebrahimi Valojerdi A: Predictive and explanatory factors of cardiovascular disease in people with adequately controlled type 2 diabetes. European journal of preventive cardiology 2017, 24(11):1181-1189.

30. Yang SH, Du Y, Li XL, Zhang Y, Li S, Xu RX, Zhu CG, Guo YL, Wu NQ, Qing P et al: Triglyceride to High-Density Lipoprotein Cholesterol Ratio and Cardiovascular Events in Diabetics With Coronary Artery Disease. The American journal of the medical sciences 2017, 354(2):117-124.

31. Lee MY, Hsiao PJ, Huang YT, Huang JC, Hsu WH, Chen SC, Shin SJ: Greater HbA1c variability is associated with increased cardiovascular events in type 2 diabetes patients with preserved renal function, but not in moderate to advanced chronic kidney disease. PloS one 2017, 12(6):e0178319.
